# Supplementary material for: A Likelihood Approach for Real-Time Calibration of Stochastic Compartmental Epidemic Models
Source: PLoS Comput Biol. 2017 Jan 17;13(1):e1005257. doi: 10.1371/journal.pcbi.1005257 (PMC5240920; doi:10.1371/journal.pcbi.1005257)
Supplement: S1 File — (TAR.GZ) [file pcbi.1005257.s014.tar.gz › HSPH_Online-SI-Revision/Additions/IPoi10/n100-extreme_noisy/n100-extreme_noisy_BENCH_table-4.pdf]

1	R0	{2.76052, 16.3913}	{2.5684, 13.7324}	{{2.5684, 13.7324}, {2.5684, 13.7324}}	{6.95956, 16.2214}	inf	10 419.	7070.29	{1244., 14 158.}	47.7176	0.37413	newly	5869.58	4721.2	{957., 8876.}	44.876	0.314654	Reff	2.28524	2.24854	{1.91133, 2.5139}	8.00968	ar	92 090.	90 114.6	{89 889., 90 310.}	2.14506
2	R0	{2.18202, 15.1734}	{2.86095, 19.9929}	{{2.86095, 19.9929}, {2.86095, 19.9929}}	{31.1147, 31.7628}	inf	10 970.	22 815.9	{12 854., 28 211.}	112.816	0.323457	newly	5914.	8279.04	{5635., 9818.}	47.4577	0.176122	Reff	1.6841	1.41445	{0.699731, 2.24665}	32.6254	ar	84 264.	93 013.9	{92 861., 93 158.}	10.384
3	R0	{2.57891, 12.5971}	{2.7346, 14.003}	{{2.7346, 14.003}, {2.7346, 14.003}}	{6.03705, 11.1605}	inf	8525.	11 117.7	{2134., 21 438.}	66.1277	0.2971	newly	6571.	6993.69	{1595., 12 008.}	45.6433	0.253169	Reff	2.202	2.19955	{1.63535, 2.6414}	12.6204	ar	90 302.	91 896.7	{91 738., 92 063.}	1.76599
4	R0	{2.5856, 9.28508}	{2.94202, 12.5548}	{{2.80569, 11.6897}, {2.9884, 12.8326}}	{14.1087, 35.286}	inf	2564.	804.806	{101., 1762.}	71.6318	0.74171	newly	3492.	784.667	{102., 1673.}	77.5765	0.870955	Reff	2.47933	2.90614	{2.7753, 2.98359}	17.3419	ar	90 312.	93 584.9	{92 650., 94 079.}	3.70699
5	R0	{2.4406, 12.1341}	{2.45621, 12.3308}	{{2.4534, 12.3139}, {2.4534, 12.3139}}	{0.639679, 1.62098}	inf	4472.	4976.26	{784., 10 464.}	70.6916	0.36658	newly	3644.	3809.74	{708., 7760.}	62.5776	0.342072	Reff	2.2517	2.23349	{1.98524, 2.4192}	6.4802	ar	88 597.	88 621.8	{88 419., 88 791.}	0.124664
6	R0	{2.72155, 15.4094}	{2.58655, 14.3523}	{{2.58655, 14.3523}, {2.58655, 14.3523}}	{4.96041, 6.8601}	inf	8392.	13 373.4	{2699., 22 123.}	86.3856	0.308576	newly	5118.	7602.59	{1895., 11 466.}	72.25	0.271983	Reff	2.34317	1.91452	{1.37923, 2.47083}	19.9378	ar	91 682.	90 338.1	{90 130., 90 562.}	1.46583
7	R0	{2.63781, 14.3935}	{2.64334, 17.2462}	{{2.64334, 17.2462}, {2.64334, 17.2462}}	{0.209644, 19.8194}	inf	9079.	6296.13	{579., 12 162.}	51.3573	0.422285	newly	5875.	3242.47	{345., 6025.}	50.0216	0.450754	Reff	2.22919	2.3587	{2.04843, 2.61664}	9.8695	ar	91 192.	90 974.9	{90 779., 91 171.}	0.249455
8	R0	{2.82391, 16.104}	{2.58655, 14.3523}	{{2.58655, 14.3523}, {2.58655, 14.3523}}	{8.40537, 10.8774}	inf	8948.	8718.67	{1043., 18 021.}	60.8166	0.393133	newly	5170.	5277.23	{757., 10 147.}	58.4997	0.356471	Reff	2.41478	2.17662	{1.66589, 2.53624}	12.4671	ar	92 664.	90 298.7	{90 134., 90 455.}	2.55254
9	R0	{2.80645, 14.6457}	{2.96978, 18.8857}	{{2.96978, 18.8857}, {2.96978, 18.8857}}	{5.81981, 28.9505}	inf	5809.	3878.17	{549., 10 098.}	61.4271	0.488246	newly	4055.	1952.05	{277., 4904.}	60.2988	0.563036	Reff	2.54301	2.79026	{2.4953, 2.94605}	10.8153	ar	92 567.	93 826.2	{93 674., 93 932.}	1.36033
10	R0	{1.86213, 5.4271}	{2.42946, 9.81882}	{{2.25463, 8.72462}, {2.6266, 11.0527}}	{30.4665, 80.922}	inf	1977.	1721.8	{106., 3958.}	57.3009	0.404799	newly	4393.	2029.88	{160., 4628.}	59.1481	0.560222	Reff	1.77612	2.36875	{2.14291, 2.60191}	33.3669	ar	75 362.	88 138.	{85 249., 90 887.}	16.9529
11	R0	{2.27349, 14.6338}	{2.37094, 13.2672}	{{2.36011, 13.1698}, {2.36011, 13.1698}}	{4.2864, 10.2697}	inf	10 491.	13 565.5	{3409., 20 969.}	61.7447	0.273949	newly	6044.	7890.12	{2480., 11 244.}	56.3257	0.243566	Reff	1.80677	1.67309	{1.15199, 2.21282}	19.7034	ar	85 805.	87 468.	{87 057., 87 559.}	1.93816
12	R0	{2.77261, 14.1894}	{2.64334, 17.2462}	{{2.64334, 17.2462}, {2.64334, 17.2462}}	{4.66239, 21.5428}	inf	6492.	3662.94	{797., 7816.}	54.6132	0.44013	newly	4508.	1977.89	{428., 4155.}	58.3654	0.507628	Reff	2.48165	2.48213	{2.28369, 2.60771}	3.99669	ar	92 508.	90 963.7	{90 789., 91 123.}	1.66942
13	R0	{2.65402, 19.3935}	{2.86095, 19.9929}	{{2.86095, 19.9929}, {2.86095, 19.9929}}	{7.79685, 3.09073}	inf	11 250.	16 731.6	{3711., 27 148.}	75.3393	0.287149	newly	4926.	6573.67	{1775., 9649.}	58.7441	0.244886	Reff	2.14402	1.97218	{1.28213, 2.69401}	20.8887	ar	91 184.	93 013.8	{92 863., 93 138.}	2.00674
14	R0	{1.75297, 4.45354}	{2.97057, 19.0921}	{{2.96978, 18.8857}, {2.97157, 19.3547}}	{69.4591, 328.694}	inf	699.	964.268	{154., 2301.}	92.0631	0.404714	newly	1855.	503.592	{77., 1225.}	73.0587	0.805235	Reff	1.72278	2.92695	{2.86703, 2.96304}	69.8964	ar	71 263.	93 834.8	{93 683., 93 987.}	31.674
15	R0	{1.99928, 7.1501}	{2.71733, 13.862}	{{2.7346, 14.003}, {2.7346, 14.003}}	{35.9154, 93.8712}	inf	3389.	3783.47	{1109., 8601.}	65.7509	0.323547	newly	4922.	2780.09	{860., 5915.}	51.9041	0.422177	Reff	1.85853	2.54663	{2.32911, 2.68565}	37.0239	ar	79 602.	91 648.5	{91 591., 92 034.}	15.1334
16	R0	{2.67398, 10.3134}	{2.67887, 14.3331}	{{2.66419, 14.2056}, {2.76203, 15.0558}}	{0.805129, 38.9758}	inf	4133.	4793.08	{968., 8184.}	60.5984	0.297348	newly	4823.	3251.52	{735., 5555.}	45.1579	0.339387	Reff	2.49563	2.46722	{2.29933, 2.65392}	4.73981	ar	91 289.	91 367.5	{91 009., 92 182.}	0.296206
17	R0	{2.44634, 16.3226}	{2.64334, 17.2462}	{{2.64334, 17.2462}, {2.64334, 17.2462}}	{8.05285, 5.65841}	inf	10 530.	10 702.2	{1982., 19 839.}	49.4698	0.288589	newly	5419.	5184.46	{1142., 8880.}	42.2204	0.260375	Reff	1.9738	2.13258	{1.65145, 2.56015}	16.0356	ar	88 710.	90 983.6	{90 807., 91 148.}	2.56291
18	R0	{2.02168, 10.1676}	{2.64334, 17.2462}	{{2.64334, 17.2462}, {2.64334, 17.2462}}	{30.7497, 69.6192}	inf	4597.	6314.85	{1334., 13 721.}	81.9568	0.322375	newly	4153.	3239.39	{777., 6765.}	51.0595	0.338474	Reff	1.82331	2.35537	{2.00592, 2.58561}	29.7334	ar	80 299.	90 989.	{90 808., 91 190.}	13.3128
19	R0	{2.89969, 15.009}	{2.36011, 13.1698}	{{2.36011, 13.1698}, {2.36011, 13.1698}}	{18.6082, 12.254}	inf	6390.	4013.9	{607., 7377.}	51.1236	0.39479	newly	4254.	2859.32	{470., 5213.}	47.7006	0.35723	Reff	2.60468	2.18814	{2.00135, 2.32915}	15.9917	ar	93 349.	87 243.2	{87 014., 87 485.}	6.54079
20	R0	{2.68933, 7.34928}	{2.73871, 14.8819}	{{2.66419, 14.2056}, {2.76203, 15.0558}}	{2.70527, 102.495}	inf	478.	252.607	{20., 576.}	56.0429	0.565353	newly	1059.	182.902	{16., 401.}	82.7288	1.02467	Reff	2.66728	2.7288	{2.65726, 2.76043}	2.99396	ar	91 574.	91 910.9	{91 138., 92 323.}	0.686643
21	R0	{2.15352, 9.28074}	{2.7346, 14.003}	{{2.7346, 14.003}, {2.7346, 14.003}}	{26.9828, 50.8824}	inf	3961.	3767.88	{478., 7323.}	65.2186	0.403173	newly	4282.	2678.	{367., 5242.}	55.1712	0.458928	Reff	1.99267	2.56553	{2.40344, 2.71343}	28.8598	ar	83 845.	91 909.9	{91 753., 92 082.}	9.61887
22	R0	{2.21953, 9.04387}	{2.7943, 13.5962}	{{2.7943, 13.5962}, {2.7943, 13.5962}}	{25.896, 50.3361}	inf	3707.	2383.05	{272., 5373.}	59.3678	0.481562	newly	4322.	1883.63	{232., 4217.}	60.6797	0.576643	Reff	2.06581	2.68884	{2.55488, 2.7822}	30.1595	ar	84 856.	92 452.	{92 284., 92 613.}	8.95162
23	R0	{2.21987, 14.5347}	{2.81635, 19.898}	{{2.81635, 19.898}, {2.81635, 19.898}}	{26.87, 36.9}	inf	10 313.	16 543.5	{5242., 24 973.}	79.3061	0.292465	newly	5905.	6581.67	{2301., 9395.}	37.2629	0.198018	Reff	1.76493	1.9718	{1.44411, 2.57919}	21.2729	ar	85 067.	92 652.6	{92 512., 92 815.}	8.91722
24	R0	{2.84268, 17.9659}	{2.49092, 17.9391}	{{2.49092, 17.9391}, {2.49092, 17.9391}}	{12.3742, 0.149171}	inf	10 277.	9169.98	{828., 18 430.}	57.0881	0.411925	newly	5220.	4153.33	{425., 7978.}	49.7433	0.39154	Reff	2.35894	2.05543	{1.57755, 2.45557}	14.609	ar	92 836.	89 127.4	{88 919., 89 338.}	3.99483
25	R0	{2.52706, 10.3679}	{2.64334, 17.2462}	{{2.64334, 17.2462}, {2.64334, 17.2462}}	{4.60139, 66.3423}	inf	4409.	4357.63	{407., 8785.}	65.1022	0.409215	newly	4655.	2312.66	{231., 4560.}	55.3522	0.523319	Reff	2.33579	2.44954	{2.24895, 2.62632}	7.42277	ar	89 623.	90 974.2	{90 818., 91 137.}	1.50761
26	R0	{2.62835, 19.1088}	{2.64334, 17.2462}	{{2.64334, 17.2462}, {2.64334, 17.2462}}	{0.57032, 9.74734}	inf	12 549.	14 916.4	{4286., 24 258.}	52.7882	0.246055	newly	5505.	6835.62	{2397., 10 131.}	50.9669	0.223758	Reff	2.05547	1.88156	{1.29558, 2.45698}	18.6492	ar	90 908.	90 999.5	{90 850., 91 246.}	0.148886
27	R0	{2.76831, 19.4155}	{2.8833, 17.1847}	{{2.8833, 17.1847}, {2.8833, 17.1847}}	{4.1538, 11.4898}	inf	16 382.	23 186.3	{8624., 28 553.}	59.6491	0.233753	newly	6900.	9764.94	{4752., 11 588.}	54.115	0.205267	Reff	1.96727	1.41398	{0.725784, 2.48638}	38.1864	ar	92 175.	93 193.7	{93 017., 93 341.}	1.10519
28	R0	{2.96609, 16.8903}	{2.78027, 13.4891}	{{2.7943, 13.5962}, {2.7943, 13.5962}}	{6.26466, 20.1369}	inf	11 248.	8453.	{1867., 17 639.}	51.4413	0.381236	newly	6075.	5850.93	{1531., 11 165.}	51.0866	0.309224	Reff	2.44352	2.38044	{1.92807, 2.71271}	10.3399	ar	94 024.	92 263.	{92 253., 92 603.}	1.87298
29	R0	{2.17046, 14.39}	{2.7811, 19.6712}	{{2.7811, 19.6712}, {2.7811, 19.6712}}	{28.1341, 36.7005}	inf	8850.	10 686.6	{1050., 21 423.}	69.2906	0.36698	newly	5160.	4514.84	{501., 8405.}	46.5837	0.337748	Reff	1.78813	2.26293	{1.70448, 2.73338}	29.7351	ar	84 001.	92 346.7	{92 214., 92 513.}	9.9353
30	R0	{2.88772, 12.4647}	{2.73587, 10.9036}	{{2.68409, 10.6354}, {2.91946, 11.8546}}	{5.74205, 12.524}	inf	3959.	3064.58	{397., 6624.}	57.2583	0.396999	newly	3670.	3167.89	{481., 6341.}	55.3619	0.351282	Reff	2.71599	2.61166	{2.46955, 2.81118}	4.9992	ar	93 273.	91 942.8	{91 209., 93 487.}	1.53846
31	R0	{2.46831, 8.14308}	{2.69821, 10.7086}	{{2.68409, 10.6354}, {2.68409, 10.6354}}	{9.31415, 31.5049}	inf	1290.	302.73	{26., 461.}	79.4906	0.934638	newly	2181.	354.333	{37., 593.}	83.7711	1.0773	Reff	2.41433	2.68976	{2.66444, 2.68369}	11.4083	ar	88 762.	90 107.4	{91 218., 91 675.}	4.69014
32	R0	{1.93236, 7.38987}	{2.92264, 13.8398}	{{2.95209, 14.037}, {2.95209, 14.037}}	{51.2471, 87.2811}	inf	2899.	3092.37	{256., 7361.}	77.9541	0.453041	newly	4137.	2362.23	{233., 5418.}	59.2309	0.536679	Reff	1.80908	2.77571	{2.54455, 2.93919}	53.4323	ar	77 654.	93 372.1	{93 547., 93 868.}	20.2411
33	R0	{2.82251, 17.6954}	{2.66615, 14.2226}	{{2.66419, 14.2056}, {2.66419, 14.2056}}	{5.53986, 19.6254}	inf	8313.	9338.43	{2503., 18 639.}	58.9799	0.294086	newly	4307.	5833.25	{1887., 10 648.}	68.7767	0.281592	Reff	2.44362	2.22788	{1.76257, 2.55253}	11.2671	ar	92 745.	91 231.2	{91 048., 91 415.}	1.63217
34	R0	{1.87083, 12.3994}	{2.76795, 19.6041}	{{2.76795, 19.6041}, {2.76795, 19.6041}}	{47.953, 58.1052}	inf	6523.	14 420.	{3662., 25 798.}	139.439	0.379971	newly	4361.	5838.62	{1766., 9355.}	62.1497	0.257471	Reff	1.57679	2.02984	{1.31619, 2.6034}	34.8284	ar	76 282.	92 238.4	{92 083., 92 437.}	20.9177
35	R0	{2.95779, 16.3003}	{2.58655, 14.3523}	{{2.58655, 14.3523}, {2.58655, 14.3523}}	{12.5513, 11.9507}	inf	6128.	5355.11	{868., 12 754.}	57.8337	0.384714	newly	3852.	3468.38	{667., 7605.}	54.5934	0.353219	Reff	2.67834	2.34736	{1.99839, 2.54832}	12.3577	ar	93 873.	90 352.5	{90 221., 90 538.}	3.75026
36	R0	{2.18718, 14.261}	{2.58655, 14.3523}	{{2.58655, 14.3523}, {2.58655, 14.3523}}	{18.2596, 0.640208}	inf	9128.	20 212.9	{12 565., 24 637.}	130.38	0.385009	newly	5307.	9985.23	{7634., 11 893.}	96.3188	0.315885	Reff	1.78461	1.24727	{0.68541, 1.99739}	37.5447	ar	84 379.	90 347.2	{90 169., 90 576.}	7.0731
37	R0	{2.44912, 9.31118}	{2.39041, 9.66608}	{{2.25463, 8.72462}, {2.87181, 13.004}}	{9.9911, 13.6388}	inf	3260.	1815.23	{256., 3672.}	58.514	0.525846	newly	3995.	2080.49	{364., 4687.}	59.0225	0.532765	Reff	2.30876	2.34928	{2.11358, 2.81437}	9.58297	ar	88 442.	87 605.6	{85 242., 93 147.}	3.88668
38	R0	{2.05949, 5.72514}	{2.50418, 12.6479}	{{2.4534, 12.3139}, {2.7346, 14.003}}	{21.5921, 120.919}	inf	809.	503.	{55., 1280.}	66.4081	0.577466	newly	2064.	419.466	{44., 1133.}	79.6771	0.982033	Reff	2.02491	2.50306	{2.39822, 2.93105}	23.6132	ar	81 587.	89 345.5	{88 320., 93 717.}	9.50944
39	R0	{1.75505, 8.52404}	{2.73737, 17.9172}	{{2.73737, 17.9172}, {2.73737, 17.9172}}	{55.9711, 110.196}	inf	4468.	6377.84	{746., 14 097.}	89.6682	0.384054	newly	4543.	3180.25	{450., 6619.}	48.1251	0.388255	Reff	1.55847	2.44577	{2.0787, 2.70529}	56.9341	ar	70 925.	91 901.4	{91 747., 92 054.}	29.5755
40	R0	{2.24724, 13.7861}	{2.60769, 14.0372}	{{2.58092, 13.7736}, {2.688, 14.8279}}	{16.0397, 1.95723}	inf	10 101.	17 297.6	{7371., 24 158.}	81.9224	0.254199	newly	6145.	9612.61	{5012., 12 315.}	64.3173	0.213219	Reff	1.78404	1.67146	{1.03518, 2.2564}	22.5848	ar	85 257.	90 575.	{90 189., 91 514.}	6.23763
41	R0	{1.77635, 8.62406}	{2.72681, 14.7497}	{{2.66419, 14.2056}, {2.76203, 15.0558}}	{53.5062, 71.03}	inf	5330.	8072.23	{758., 17 290.}	97.3131	0.399028	newly	5334.	4933.61	{581., 9795.}	53.6864	0.357832	Reff	1.53452	2.34962	{1.89411, 2.69419}	53.221	ar	72 941.	91 838.2	{91 173., 92 314.}	25.9075
42	R0	{1.80397, 5.4932}	{2.6266, 11.0527}	{{2.6266, 11.0527}, {2.6266, 11.0527}}	{45.6011, 101.207}	inf	2276.	4286.44	{302., 9316.}	129.325	0.43701	newly	4341.	4092.52	{314., 8487.}	59.911	0.396568	Reff	1.7106	2.43652	{2.21559, 2.61418}	42.4371	ar	73 232.	90 788.6	{90 597., 90 996.}	23.974
43	R0	{2.83096, 16.7077}	{2.7811, 19.6712}	{{2.7811, 19.6712}, {2.7811, 19.6712}}	{1.76124, 17.7373}	inf	8628.	7954.38	{1439., 14 956.}	51.3705	0.33849	newly	4809.	3507.83	{660., 6458.}	43.248	0.342144	Reff	2.43862	2.41169	{2.07428, 2.71875}	8.72476	ar	92 740.	92 309.8	{92 153., 92 441.}	0.46383
44	R0	{2.73678, 11.6021}	{2.95209, 14.037}	{{2.95209, 14.037}, {2.95209, 14.037}}	{7.86727, 20.9867}	inf	6552.	8518.45	{1477., 17 230.}	73.378	0.326114	newly	6185.	5816.66	{1254., 10 800.}	48.8312	0.296391	Reff	2.44682	2.54415	{2.10661, 2.88534}	10.9667	ar	92 003.	93 725.8	{93 571., 93 861.}	1.8726
45	R0	{2.14235, 6.98601}	{2.67691, 14.3161}	{{2.66419, 14.2056}, {2.76203, 15.0558}}	{24.952, 104.926}	inf	2850.	2685.05	{143., 7286.}	75.0963	0.515151	newly	4808.	1873.19	{115., 4867.}	64.3131	0.707149	Reff	2.02463	2.5583	{2.34129, 2.66155}	26.3589	ar	82 974.	91 335.6	{91 016., 92 118.}	10.0773
46	R0	{2.23409, 9.21289}	{2.5684, 13.7324}	{{2.5684, 13.7324}, {2.5684, 13.7324}}	{14.964, 49.0564}	inf	2544.	1882.13	{136., 3844.}	59.8981	0.480709	newly	3017.	1390.85	{96., 2809.}	60.6034	0.602813	Reff	2.12542	2.48757	{2.40189, 2.56293}	17.0386	ar	85 163.	90 135.7	{89 945., 90 295.}	5.83901
47	R0	{2.12258, 8.15265}	{2.6266, 11.0527}	{{2.6266, 11.0527}, {2.6266, 11.0527}}	{23.7456, 35.5719}	inf	2945.	1997.95	{165., 3622.}	55.8852	0.46701	newly	3839.	2044.09	{180., 3654.}	56.4096	0.51777	Reff	1.99728	2.54057	{2.4685, 2.61935}	27.2013	ar	82 690.	90 772.	{90 558., 91 024.}	9.7739
48	R0	{2.71771, 10.4636}	{2.81617, 13.9425}	{{2.81617, 13.9425}, {2.81617, 13.9425}}	{3.6229, 33.2476}	inf	2061.	931.215	{61., 1911.}	66.4591	0.676062	newly	2437.	743.246	{42., 1584.}	72.2212	0.815585	Reff	2.62862	2.77529	{2.73101, 2.81369}	5.6506	ar	91 716.	92 615.5	{92 408., 92 748.}	0.980787
49	R0	{2.44369, 6.66477}	{2.74764, 10.9646}	{{2.68409, 10.6354}, {2.91946, 11.8546}}	{12.4382, 64.5156}	inf	1213.	451.677	{58., 1030.}	69.681	0.698392	newly	3032.	513.8	{75., 1176.}	83.0541	0.993574	Reff	2.39345	2.73686	{2.64815, 2.91277}	14.3481	ar	88 827.	92 056.	{91 234., 93 536.}	3.63516
50	R0	{2.77383, 17.9775}	{2.96454, 19.3116}	{{2.97157, 19.3547}, {2.97157, 19.3547}}	{6.95434, 7.44093}	inf	14 883.	14 457.1	{4378., 25 280.}	42.7918	0.244542	newly	6948.	6196.85	{2147., 9917.}	34.6651	0.213795	Reff	2.0682	2.22608	{1.59921, 2.73405}	18.4962	ar	92 334.	93 770.7	{93 695., 94 005.}	1.59861
